# Isochronous Sequential Presentation Helps Children Orient Their Attention in Time

**DOI:** 10.3389/fpsyg.2016.01417

**Published:** 2016-09-22

**Authors:** Katherine A. Johnson, Marita Bryan, Kira Polonowita, Delia Decroupet, Jennifer T. Coull

**Affiliations:** ^1^School of Psychological Sciences, University of Melbourne, ParkvilleVIC, Australia; ^2^Laboratoire des Neurosciences Cognitives, Aix-Marseille Université, CNRSMarseille, France

**Keywords:** temporal attention, spatial attention, rhythm, temporal prediction, temporal expectation, exogenous attention

## Abstract

Knowing when an event is likely to occur allows attentional resources to be oriented toward that moment in time, enhancing processing of the event. We previously found that children (mean age 11 years) are unable to use endogenous temporal cues to orient attention in time, despite being able to use endogenous spatial cues (arrows) to orient attention in space. Arrow cues, however, may have proved beneficial by engaging exogenous (automatic), as well as endogenous (voluntary), orienting mechanisms. We therefore conducted two studies in which the exogenous properties of visual temporal cues were increased, to examine whether this helped children orient their attention in time. In the first study, the location of an imperative target was predicted by the direction of a left or right spatial arrow cue while its onset was predicted by the relative duration of a short or long temporal cue. To minimize the influence of rhythmic entrainment in the temporal condition, the foreperiod (500 ms/1100 ms) was deliberately chosen so as not to precisely match the duration of the temporal cue (100 ms/400 ms). Targets appeared either at cued locations/onset times (valid trials) or at unexpected locations/onset times (invalid trials). Adults’ response times were significantly slower for invalid versus valid trials, in both spatial and temporal domains. Despite being slowed by invalid spatial cues, children (mean age 10.7 years) were unperturbed by invalid temporal cues, suggesting that these duration-based temporal cues did not help them orient attention in time. In the second study, we enhanced the exogenous properties of temporal cues further, by presenting multiple temporal cues in an isochronous (rhythmic) sequence. Again, to minimize automatic entrainment, target onset did not match the isochronous interval. Children (mean age 11.4 years), as well as adults, were now significantly slowed by invalid cues in both the temporal and spatial dimension. The sequential, as opposed to single, presentation of temporal cues therefore helped children to orient their attention in time. We suggest that the exogenous properties of sequential presentation provide a temporal scaffold that supports the additional attentional and mnemonic requirements of temporal, as compared to spatial, processing.

## Introduction

Our attentional system allows us to filter distracting stimuli to efficiently process relevant information. Moreover, we can flexibly direct (or “orient”) attentional resources to a stimulus appearing at a specific location in space (Posner, 1980) or moment in time (Coull and Nobre, 1998), allowing individuals to better process that stimulus and so respond to the environment in an appropriate manner (Coull, 2009; Carrasco, 2011). The ability to orient attention in space has a long and considerable research history, with the research of Wundt, James, and Helmholtz in the 19th century (Carrasco, 2011) providing a platform for more recent experimental research (Posner, 1980; Enns and Brodeur, 1989; Corbetta and Shulman, 2002; Posner, 2016). The ability to orient attention in time using similar experimental paradigms is a newer research area, with the vast majority of research being conducted with adult participants (Coull and Nobre, 1998; Correa et al., 2004; Nobre et al., 2007). Nevertheless, a previous developmental study of our own, which examined attention in both time and space with valid and invalid cues, has shown that children have difficulties using a symbolic cue to voluntarily orient their attention in time, but can use an arrow cue to orient their attention in space (Johnson et al., 2015). The spatial properties of the arrow space cue, however, may have induced automatic, in addition to voluntary, attentional mechanisms (Ristic and Kingstone, 2009), making the time and space cues unbalanced. Two studies are presented in which we manipulated the temporal properties of the time cues to test whether this would help children to orient their attention in time.

In the spatial domain, two partially segregated attentional orienting systems in the human brain – the exogenous and endogenous systems – have been extensively described (Corbetta et al., 2002). The exogenous spatial orienting system directs attention automatically to salient stimuli in the environment. In a typical exogenous spatial orienting task, a cue is presented in a peripheral location. A target then appears, either in the same location as the cue (valid trial) or in the non-cued location (invalid trial), and the participant must respond as quickly as possible to the appearance of the target (Posner, 1980). Responses are quicker to targets appearing in the cued than the invalidly cued spatial location. This “validity effect” is observed even when the location of the cue does not necessarily predict where the target will eventually appear (Ristic et al., 2002; Tipples, 2002), attesting to the automatic nature of the spatial orienting mechanism. The endogenous orienting system, on the other hand, directs attention through goal-directed voluntary mechanisms, based on knowledge and expectation (Corbetta et al., 2002). In a typical endogenous spatial orienting paradigm, attention is voluntarily directed to one location or another in response to symbolic or abstract centrally presented cues (e.g., arrows) that provide information about where the upcoming target is likely to appear (Posner, 1980). Again, RTs are faster to targets appearing in validly cued, rather than invalidly cued, locations. While the exogenous orienting system operates from infancy (Harmon et al., 1994; Richards, 2000), the use of endogenous cues to voluntarily orient attention in space develops later, around 6–8 years (Pearson and Lane, 1990; Rueda et al., 2004; Wainwright and Bryson, 2005; Iarocci et al., 2009).

The endogenous spatial orienting paradigm has now been adapted for the temporal domain, with symbolic temporal cues predicting when, rather than where, the target would appear (Coull and Nobre, 1998). Temporally predictive cues allowed attention to be endogenously oriented toward the predicted moment in time, with RTs being faster for validly, rather than invalidly, cued targets (Coull et al., 2000; Correa et al., 2004, 2010). Despite the growing number of studies in adults, only a handful of studies have investigated whether children can use endogenous temporal cues to orient their attention in time. Two very recent studies by Mento and Tarantino (2015) and Mento and Vallesi (2016) have shown that children (aged 6–12) can use symbolic temporal cues to endogenously orient their attention in time. When we compared spatial and temporal orienting directly in our own study, however, children (average age 11 years) had difficulties using a symbolic cue to orient their attention in time, even though they could use an arrow cue to orient their attention in space (Johnson et al., 2015). We concluded that the development of endogenous temporal orienting lags behind that of endogenous spatial orienting. The symbolic spatial cues used in the Johnson et al. (2015) study were left or right-facing arrows. Arrows, though symbolic and therefore assumed to orient spatial attention endogenously, have actually been shown to also induce exogenous orienting of attention (Eimer, 1997; Tipples, 2002; Ristic and Kingstone, 2009; Olk, 2014). The relative benefits of spatial, over temporal, cues in Johnson et al.’s (2015) study might have been due, therefore, to the additional exogenous orienting mechanisms induced by the arrow stimuli. By contrast, Johnson et al.’s (2015) temporal cues were more purely endogenous in nature: short and long lines represented short or long temporal intervals. There is evidence that children as young as 4 or 5 represent time in spatial terms, with the spatial length of a stimulus biasing temporal estimates of its duration (Casasanto et al., 2010; Bottini and Casasanto, 2013). It is therefore possible that short/long lines could induce exogenous, as well as endogenous, orienting of attention to short/long temporal intervals. This hypothesis has not yet, to our knowledge, been formally tested. In the absence of evidence, the possibility remains that the developmental lag for temporal, versus spatial, orienting reported by Johnson et al. (2015) was not due to the spatial versus temporal nature of the cues, but rather to their differential capacity for inducing exogenous and endogenous attentional mechanisms.

We therefore decided to compare temporal and spatial orienting in children using symbolic cues that were hypothesized to induce exogenous, as well as endogenous, mechanisms in both temporal and spatial domains. Just as the physical location of a stimulus can act as an exogenous spatial cue, the physical duration of a stimulus can act as an exogenous temporal cue. Indeed the constant duration of the intervals delineating an isochronous (rhythmic) stimulus sequence guides temporal attention to moments in time that are in phase (on beat) with the temporal structure of the sequence, without the need for attentional instruction (Klein and Jones, 1996; Large and Jones, 1999; Jones et al., 2002; Rohenkohl et al., 2011; Sanabria et al., 2011; Triviño et al., 2011; Bolger et al., 2014). The temporal predictability of isochronous sequences improves sensorimotor processing of events occurring in phase with the rhythm, both enhancing perceptual sensitivity (Barnes and Jones, 2000; Jones et al., 2002; Lange et al., 2003; Morillon et al., 2014) and speeding visual target detection (Coull and Nobre, 2008; Bolger et al., 2014). As such, the temporal properties of stimulus presentation may be considered as an exogenous temporal cue. Like exogenous spatial cues, exogenous temporal cues can orient attention automatically. For example, Sanabria et al. (2011) showed that RTs to targets presented in phase with the temporal rhythm were faster even though the target was equally likely to appear out of phase with the rhythm (Sanabria et al., 2011). Similarly, responses were faster to in-phase targets even when participants were not required to attend to the rhythmic sequence (Rohenkohl et al., 2011; Breska and Deouell, 2014) or when participants had to simultaneously perform a demanding secondary working memory task (de la Rosa et al., 2012; Cutanda et al., 2015).

Like exogenous spatial orienting, exogenous temporal orienting also appears to operate from infancy. Infants detect unexpected temporal patterns (Brannon et al., 2004, 2007; vanMarle and Wynn, 2006), and can use rhythm to create temporal expectancies (Haith et al., 1988; Colombo and Richman, 2002; Philips-Silver and Trainor, 2005; Bergeson and Trehub, 2006; Werner et al., 2009; Winkler et al., 2009; Brandon and Saffran, 2011). These studies suggest that infants have temporally predictive information processing capabilities (Trainor, 2012). From around the age of 4 years, children can tap in time with isochronous, rhythmic, and musical sequences, and can discriminate between the tempos of two drum sequences (Drake et al., 2000). Moreover, older children (around 11 years) can use temporal patterns to predict in time when a target will appear, allowing them to respond more quickly to that target (Durston et al., 2007). In sum, children are able to build up a temporal expectancy based on the temporal properties of sensory input, which directs their attention exogenously in time.

The two new studies reported here were designed to test the prediction that children would be able to benefit from a temporal cue when the cue conveyed temporal information in a more exogenous manner. In the first study, temporal information was conveyed by the actual presentation duration of the temporal cue: the cue was presented for either 100 or 400 ms to indicate that the target would appear after a short (500 ms) or a long (1100 ms) delay. In the second study, temporal information was again conveyed by the duration of the cue, though this time the cue was presented five times in a row to reinforce the representation of cue duration.

## Study 1: Temporal Information Conveyed By Cue Duration

In the Johnson et al. (2015) study both the space and time cues were presented for 100 ms, a presentation time typical of these types of cognitive studies (Coull and Nobre, 1998). The presentation duration of the cue, however, can itself convey temporal information about when the target is expected to appear in a bottom-up, or exogenous, manner. Presenting the temporal cue for either a long (e.g., 400 ms) or a short (e.g., 100 ms) duration to reflect the upcoming period between the cue and the target’s appearance (the foreperiod, “FP”) may help the participant to extract temporally pertinent information from the cue’s physical appearance. While the temporal cue used in this study retained the visual qualities of our previous paradigm (Johnson et al., 2015)– it was an abstract symbol comprised of thick or thin lines that predicted a long (1100 ms) or short (500 ms) FP, respectively – we additionally incorporated temporal information in a more exogenous manner, by manipulating the duration of cue presentation. It is important to note that the presentation duration of the temporal cue was not equal to the upcoming FP; participants had to estimate the *relative* duration of the cue (short/long) and apply this information to a new set of timing parameters, in order to predict when (soon/later) the target would occur.

Children in the age range tested in this study are able to estimate the duration of stimuli as accurately as adults (Droit-Volet and Wearden, 2001; McCormack et al., 2005; Droit-Volet and Coull, 2015; Droit-Volet, 2016). For instance, 10-year-old children performed similarly to adults on two variants of the temporal generalization task, in which participants were presented with a pair of stimuli and asked to judge whether they were of the same duration (McCormack et al., 2005). In the temporal bisection task participants are trained to recognize two stimulus durations as either “short” or “long,” and are then tested on a range of probe durations and asked to decide whether a probe is either short or long. Children as young as 3 years of age are able to complete this task with orderly data, demonstrating an ability to process temporal information albeit less accurately than 5- or 8-year-olds (Droit-Volet and Wearden, 2001), while 10-year-olds perform as well as adults (Droit-Volet and Coull, 2015). We were therefore confident that the 10- to 12-year-olds in our study would be able to accurately time the duration of the time cue.

The aim of study 1 was to investigate if children would be able to use the physical duration of the time cue to predict when the target would appear so as to speed response times (RTs). The hypothesis was that children and adults would show the validity effect for both the time and space cues, with faster responses to validly versus invalidly cued trials.

### Materials and Methods

#### Participants

Thirty-three typically developing children (20 female) and 30 adults (20 female) participated in the study. Thirteen children (eight female) were excluded as they made over 50 omission errors on the task, suggesting task disengagement. Please note that the outcome of the results remained the same when these participants were included in the sample. The final sample consisted of 20 children (12 female) and 30 adults (20 female). The children ranged in age from 10 to 12 years (mean 10.7, *SD* 0.8); the adults ranged in age from 18 to 23 years (mean 19.2, *SD* 1.3). The children were recruited from two primary schools in Melbourne, Victoria. The adults were recruited from the University of Melbourne first year cohort of Psychology students via a Research Experience Program, for which they received course credit. Children’s estimated full-scale intelligence quotient (IQ) was calculated using the WISC-IV (Wechsler, 2004); 16 children completed the four subtest assessment using Block Design, Similarities, Digit Span and Coding, whilst four children completed the two subtest assessment using Block Design and Vocabulary. The children’s estimated full scale IQs were calculated using Sattler’s method (Sattler and Dumont, 2004), and all scored above 70 (mean 105, *SD* 10, range 81–121).

The University of Melbourne Human Research Ethics Committee and the Catholic Education Office in the Archdiocese of Melbourne approved the study, in accordance with the 1964 Declaration of Helsinki. Parents and children provided written informed consent prior to each child’s participation in the study. Adult participants provided written informed consent prior to the study.

#### Experimental Task

**FIGURE 1:**
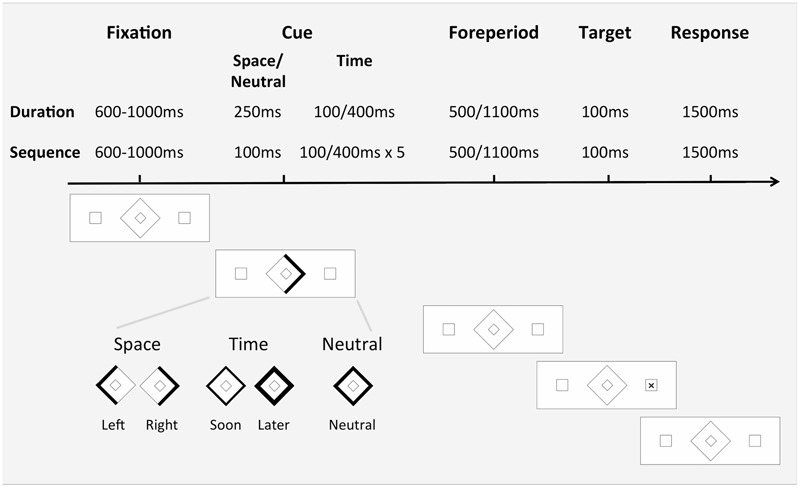
**Sequence of events in one trial (right spatial, valid) and examples of the Cue stimuli used in the three orienting conditions.** In the Space trials, either the left- or right-hand side of the stimuli thickened slightly, indicating the likely appearance of the target in the left or right peripheral box, respectively. In the neutral condition, the outline of the central stimulus thickened slightly but did not provide any specific information about the likely location or FP of the target and so simply alerted the participant to the upcoming target. In Study 1 in the duration time condition, the outline of the central stimulus thickened either very slightly for 100 ms or to a much greater extent for 400 ms, indicating that the target was likely to appear soon (500 ms FP) or later (1100 ms FP), respectively. In Study 2 in the sequential time condition, for the short FP the background stimulus display was shown for 100 ms and then the outline of the central stimulus thickened very slightly for 100 ms. This off/on cycle occurred five times in a row, to indicate that the target was likely to appear soon. For the long FP, the background stimulus display appeared for 100 ms, then a thick outline of the central stimulus appeared for 400 ms. This off/on cycle occurred five times in a row, signaling that the target was going to appear later.

All participants completed a modified version of the spatial and temporal orienting task (Coull and Nobre, 1998), which was presented using E-prime Software (Psychology Software Tools) on a 15-inch laptop computer. The modification comprised the use of a duration-based temporal cue. Participants were presented with a central stimulus display containing a central diamond and two peripheral boxes (**Figure 1**). The participants were asked to maintain their gaze on the central stimulus and use the information presented there to help predict the appearance of the upcoming target, an ‘x’, in one of the two peripheral boxes. The aim of the participants was to respond as quickly as possible to the appearance of the target, by pressing the down arrow on the computer keyboard. The participants simply needed to detect the appearance of the target.

Three Cue conditions were presented in separate blocks, the order of which was counterbalanced across participants (**Figure 1**). Within a trial, participants were initially exposed to the background stimulus display for a 600, 700, 800, 900, or 1000 ms inter-trial interval, randomized across trials. In the space condition, the line comprising the left or right side of the central stimulus thickened slightly for 250 ms, indicating the likely appearance of the target in the left or right peripheral box, respectively. In the time condition, the outline of the central stimulus thickened either very slightly for 100 ms or to a much greater extent for 400 ms, indicating that the target was likely to appear soon (500 ms FP) or later (1100 ms FP), respectively. In the neutral condition, the outline of the central stimulus thickened slightly for 250 ms but did not provide any specific information about the likely location or FP of the target and so simply alerted the participant to the upcoming target. For all Cue conditions the background stimulus display then remained unchanged for a FP of either 500 or 1100 ms. The timing of the FP started with the offset of the cue. The target then appeared in either the left or right peripheral box for 100 ms. Following target presentation, the background stimulus display was shown for 1500 ms, to allow for participant’s responses, before the next trial commenced.

For the space and time conditions, 32 valid, 8 invalid, and 4 catch trials (44 trials in total) were presented in each of three consecutive blocks (132 trials per condition). For the neutral condition, 16 trials were presented in each of three consecutive blocks (48 trials). Prior to each block, participants were informed of the nature of the cue in the upcoming block. Each block lasted for between 2 to 3 min and participants were able to take rest breaks between blocks. The whole task, with breaks, took approximately 20–25 min.

Participants were provided with a training set of 32 valid trials for the space and time conditions, and 16 trials for the neutral condition, prior to the experimental session. This was to ensure they understood the instructions and, for the time condition, to learn the association between the duration cues and the short and long FPs. The spatial and temporal cues were trained for the same number of trials so that they were subject to the same degree of learning-induced transfer from endogenous to exogenous attention control (Lin et al., 2016). The participants were asked to identify the meaning of each of the cues to ensure understanding of the cues. They were reminded to respond to target appearance as quickly as possible.

#### Procedure

The children were tested in a quiet setting at their schools. The adults were tested in a quiet testing room in the School of Psychological Sciences at the University of Melbourne.

#### Data Analysis

RTs of less than 100 ms (errors of omission, extremely fast RTs) were excluded from the RT analyses (see **Table 1** for a count of omission errors). Any RTs to the catch trials were also excluded. For each participant, the mean RT was calculated per trial type, and group means (M) and standard deviations (SDs) were then calculated. The data were normally distributed.

**Table 1:** The median and interquartile range (in parentheses) measures of the number of omission errors, for the Adult and Child groups for Study 1 (duration time cue) and Study 2 (sequence time cue) on the various levels of the Cue (space, time), Validity (valid, invalid), and Foreperiod (500 ms, 1100 ms) independent variables of the spatial and duration temporal orienting task.

Study	Group		Space Valid 500 ms	Space Invalid 500 ms	Space Valid 1100 ms	Space Invalid 1100 ms	Time Valid 500 ms	Time Invalid 500 ms	Time Valid 1100 ms	Time Invalid 1100 ms	Neutral 500 ms	Neutral 1100 ms
Duration	Adults	Median and IQR	0.5 (2)	0.0 (0)	1.0 (3)	0.0 (1)	1.0 (2)	0.0 (1)	1.0 (2)	0.0 (0)	0.0 (1)	0.0 (2)
Duration	Children	Median and IQR	3.5 (3)	1.0 (2)	4.0 (7)	1.0 (2)	2.0 (4)	0.0 (1)	2.5 (4)	1.5 (3)	1.0 (2)	1.0 (5)
Sequence	Adults	Median and IQR	0.0 (2)	0.0 (0)	1.0 (1)	0.0 (1)	0.0 (1)	0.0 (0)	0.0 (1)	0.0 (1)	0.0 (0)	0.0 (1)
Sequence	Children	Median and IQR	2.0 (2)	1.0 (2)	2.0 (4)	0.0 (1)	3.0 (4)	0.0 (1)	1.0 (3)	0.0 (1)	0.0 (1)	1.0 (4)

#### Statistics

Statistical analysis was carried out using IBM SPSS software version 23. The validity effect was investigated with a three-way mixed factorial ANOVA with Group (adults, children) as a between-subjects factor and Cue type (space, time) and Validity (valid, invalid) as within-subjects factors. The validity effect was calculated using data from the 500 ms FP trials only to avoid confounding the temporal validity effect with Variable FP effects. In cued RT paradigms, the probability of target appearance increases with the length of the FP – the “Hazard Function” (Luce, 1986) – which leads to faster RTs at longer FPs (Woodrow, 1914) – the “Variable FP effect” (Niemi and Näätänen, 1981). In temporal orienting paradigms, the RT benefits of the Hazard Function render the RT benefits of the temporally valid cue negligible at long FPs (Coull and Nobre, 1998; Coull et al., 2000; Vallesi et al., 2013). To obtain a clean measure of temporal orienting effects, we therefore constrained our analysis to the short (500 ms) data-point (Rohenkohl et al., 2011, for a similar approach). Refer to **Table 2** for data for the 1100 ms condition.

**Table 2:** Mean and standard deviation (in parentheses) measures of response time, in milliseconds, for the Adult and Child groups for Study 1 (duration time cue) and Study 2 (sequence time cue) on the various levels of the Cue (space, time), Validity (valid, invalid), and Foreperiod (500 ms, 1100 ms) independent variables of the spatial and duration temporal orienting task.

Study	Group	Space Valid 500 ms	Space Invalid 500 ms	Validity effect	Space Valid 1100 ms	Space Invalid 1100 ms	Validity effect	Time Valid 500 ms	Time Invalid 500 ms	Validity effect	Time Valid 1100 ms	Time Invalid 1100 ms	Validity effect
Duration	Adults	302 (48)	334 (53)	32	291 (38)	315 (42)	24	284 (54)	312 (51)	28	296 (42)	304 (48)	8
Duration	Children	340 (57)	373 (69)	33	340 (51)	355 (59)	15	344 (52)	344 (54)	0	337 (46)	338 (51)	1
Sequence	Adults	304 (44)	336 (55)	32	296 (35)	318 (44)	22	298 (51)	333 (51)	35	284 (35)	313 (37)	29
Sequence	Children	352 (65)	407 (80)	54	333 (58)	356 (60)	23	335 (77)	416 (82)	81	343 (80)	357 (79)	14

Data from the neutral condition was used to calculate the Variable FP effect and the sequential effect. The sequential effect reflects the fact that a participant’s RT to the upcoming target depends on the duration of the FP of the previous trial (Woodrow, 1914; Karlin, 1959; Baumeister and Joubert, 1969). Responses are slower on short FP trials when the previous trial had a long FP; in contrast responses for long FP trials are not influenced by the previous trial’s FP (Los and van den Heuvel, 2001; Los and Heslenfeld, 2005). The sequential effect is thought to be an automatic form of temporal prediction (Los and van den Heuvel, 2001; Triviño et al., 2011; Vallesi et al., 2013, 2014). The variable FP and sequential effects were investigated with a three-way mixed factorial ANOVA involving Group (adults, children), FP of the current trial, i.e., FP(*n*) (500 ms, 1100 ms), and FP of the previous trial, i.e., FP(*n* – 1) (500 ms, 1100 ms). The FP and sequential effects were investigated using the neutral trials only, to avoid confounds from any effects associated with the space and time cues. The alpha level was set at 0.05 and Bonferroni-adjustments were made for pair-wise comparisons.

### Results

#### Spatial and Temporal Validity effects

**FIGURE 2:**
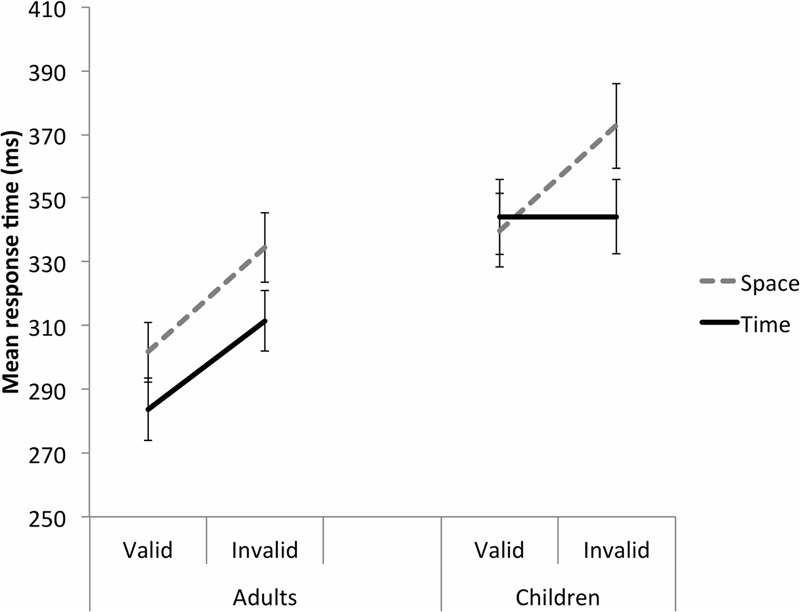
**Study 1 (duration time cue).** A Group by Cue by Validity interaction for the Validity effect. Adults were perturbed by the invalid trials in both the space and time cue conditions. In contrast, children were only perturbed by the invalid trials in the space condition. Error bars reflect standard errors.

Significant Group *F*(1,48) = 8.648, *p* = 0.005, $\eta_{p}^{2}$ = 0.153, Cue *F*(1,48) = 13.354, *p* = 0.001, $\eta_{p}^{2}$ = 0.218, and Validity *F*(1,48) = 57.741, *p* < 0.001, $\eta_{p}^{2}$ = 0.546 main effects were further explained by a significant Group by Cue by Validity interaction, *F*(1,48) = 5.828, *p* = 0.020, $\eta_{p}^{2}$ = 0.108 (**Figure 2**; **Table 2**). This was broken down by Group. For the adults, there was a significant Cue main effect, *F*(1,29) = 15.137, *p* = 0.001, $\eta_{p}^{2}$ = 0.343, such that adults responded significantly more quickly to the time than space cues. There was also a significant Validity main effect, *F*(1,29) = 57.627, *p* < 0.001, $\eta_{p}^{2}$ = 0.665, whereby adults responded significantly more quickly to the valid than invalid trials. There was no significant Cue by Validity interaction, *F*(1,29) = 0.431, *p* = 0.517, $\eta_{p}^{2}$ = 0.015, suggesting valid cues were equally beneficial in space and time. For the children, on the other hand, there was a significant Cue by Validity interaction, *F*(1,19) = 15.379, *p* = 0.001, $\eta_{p}^{2}$ = 0.447. On the space trials, children responded significantly more quickly to the valid than invalid trials, *p* < 0.001. On the time trials, however, there was no significant different in MRT between the valid and invalid trials, *p* = 0.988. On valid trials there was no significant difference in MRT between the space and time cues, *p* = 0.519. On invalid trials, the children responded significantly more slowly to the space cues than the time cues, *p* = 0.014.

#### Sequential and Variable Foreperiod Effects

**FIGURE 3:**
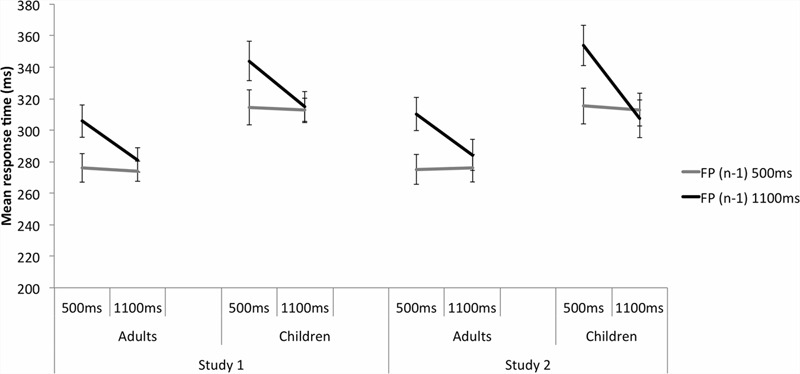
**A significant FP(*n*) × FP(*n* - 1) interaction in the Neutral trials for Study 1 on the left and Study 2 on the right.** This indicates the presence of the sequential effect, in both adults and children equally. Participants responded more slowly when the current trial’s FP was short and was preceded by a long FP trial, compared with a preceding short FP trial. Responses did not vary significantly when the current long FP trial was preceded by a long or short FP. Results were very similar across studies 1 and 2. Error bars reflect standard errors.

Significant FP, *F*(1,48) = 5.711, *p* = 0.021, $\eta_{p}^{2}$ = 0.106, and FP(*n* - 1), *F*(1,48) = 21.774, *p* < 0.001, $\eta_{p}^{2}$ = 0.312, were further explained by a significant FP by FP(*n* - 1) interaction, *F*(1,48) = 19.422, *p* < 0.001, $\eta_{p}^{2}$ = 0.288 (**Figure 3**; **Table 3**). At the 500 ms FP, participants were significantly faster to respond to targets when the previous trial’s FP was also 500 ms rather than 1100 ms, *p* < 0.001, reflecting the sequential effect. At 1100 ms FP, there was no significant difference in RT between the FP(*n* - 1) 500 ms and FP(*n* - 1) 1100 ms trials, *p* = 0.274, reflecting the asymmetrical nature of the sequential effect. When the previous trial’s FP was short, participants responded with similar RTs between the 500 and 1100 ms FP trials, *p* = 0.749. When the previous trial’s FP was long, participants were significantly slower to respond to the target at 500 ms FP compared with the 1100 ms FP trials, *p* < 0.001.

**Table 3:** Mean and standard deviation (in parentheses) measures of response time, in milliseconds, for the Adult and Child groups of Study 1 (duration time cue) and Study 2 (sequence time cue), on the Neutral trials, on the various levels of the previous trial foreperiod FP(*n* - 1) (500 ms, 1100 ms) and the current trial FP(*n*) (500 ms, 1100 ms) of the spatial and duration temporal orienting task.

Study	Group	FP(*n* - 1) 500 ms	FP(*n* - 1) 1100 ms	FP(*n* - 1) 500 ms	FP(*n* - 1) 1100 ms
		
		FP(*n*) 500 ms	FP(*n*) 500 ms	FP(*n*) 1100 ms	FP(*n*) 1100 ms
Duration	Adults	276 (43)	306 (57)	274 (37)	281 (39)
Duration	Children	315 (59)	344 (55)	313 (33)	315 (47)
Sequence	Adults	275 (40)	310 (51)	276 (37)	284 (49)
Sequence	Children	315 (63)	354 (65)	313 (57)	307 (61)

The adults performed the task with significantly faster MRT than the children, *F*(1,48) = 11.313, *p* = 0.002, $\eta_{p}^{2}$ = 0.191. There were no other significant interactions.

### Discussion

As expected, adults’ attention was guided by both the time and space cues: participants responded with a significantly slower MRT to invalidly cued targets in both dimensions. The results, however, failed to support our hypothesis that manipulating the duration of the temporal cue would enable temporal orienting in children. We found that children were not significantly perturbed by the invalid time cues, suggesting that they were not using the duration cue to anticipate when the target would appear. In contrast, presenting the children with the invalid space cue did result in a significantly longer RT compared to the valid space cue, suggesting the children were using the space cues to anticipate *where* the target would appear. Moreover, the children demonstrated the sequential and variable FP effects, supporting previous results (Vallesi and Shallice, 2007; Johnson et al., 2015; Mento and Tarantino, 2015), indicating that children implicitly processed the temporal information available in the trial structure. Their responses were faster to the targets in the long FP trials, reflecting the variable FP effect, and their responses were slower when the preceding trial’s cue-target interval was longer than that of the current trial, reflecting the sequential effect. Indeed, our data further suggest that these effects were even stronger in children compared with the adults (Johnson et al., 2015).

Overall, our results suggest that the temporal information conveyed by the duration-based time cue was not enough to help children anticipate when the target would appear. Their performance was very similar to that of the children in the Johnson et al. (2015) study. Although children in this age range can estimate stimulus duration as well as adults (McCormack et al., 2005; Droit-Volet and Coull, 2015; Droit-Volet, 2016), it appears that they are not yet able to use this information to make temporal predictions in order to optimize behavior. Yet previous studies have shown that even young infants derive temporal expectations from isochronous or rhythmic sequences of stimuli (Haith et al., 1988; Colombo and Richman, 2002; Philips-Silver and Trainor, 2005; Werner et al., 2009; Winkler et al., 2009; Brandon and Saffran, 2011). In Study 2 therefore, we further increased the exogenous temporal information conveyed by the temporal cue by presenting cues in an isochronous sequence. We aimed to test whether this would help children of 10–12 years to orient attention in time in order to speed responding to a temporally predictable event.

## Study 2: Temporal Information Conveyed By An Isochronous Sequence

In this study, we presented the duration-based temporal cue several times in an isochronous sequence, in order to enhance the temporal properties of the cue. Many previous studies in adults have shown that the variability of duration estimates decreases with the number of stimulus repetitions (Keele et al., 1989; Schulze, 1989; Drake and Botte, 1993; Ivry and Hazeltine, 1995; Grondin et al., 2001; Merchant et al., 2008; Grondin, 2012). We therefore hypothesized that presenting our duration-based time cue multiple times would help children form a more robust temporal memory of cue duration, which would then help them form temporal predictions concerning target onset time. In the current study, five repetitions of the temporal cue were presented in quick succession, with either a 100 ms short on and 100 ms off cycle, or a 400 ms long on and 100 ms off cycle, indicating that the upcoming FP would be short (500 ms) or long (1100 ms), respectively. Importantly, the time of target onset was not in phase with the preceding rhythm: instead, as in Study 1, participants had to extract the relative duration of the cue and extrapolate it to a new set of timing parameters, in order to predict when the target would occur. In this way, the isochronous time cue was not simply entraining temporal attention in a purely exogenous manner (Klein and Jones, 1996; Large and Jones, 1999; Jones et al., 2002). Sanabria et al. (2011, Experiment 3) have already shown that adult participants extrapolate the temporal information provided by non-predictive rhythmic sequences to non-matching FPs in order to speed responding (Sanabria et al., 2011). The aim of study 2 was to investigate whether children could similarly use the temporal information provided by an isochronous sequence to anticipate when the target would appear. The space cue was the same as in Study 1 (arrows) and is consistent with our previous research (Johnson et al., 2015). Our hypothesis was that children and adults would show the validity effect for both the time and space cues.

### Materials and Methods

#### Participants

Twenty-four typically developing children (15 female) and 31 adults (26 female) participated in the study. Four children and one adult were excluded as they made over 50 omission errors on the task, suggesting task disengagement. One adult was excluded, as her RTs were greater than 2.5 SD above the adult group. Please note that the outcome of the results remained the same when these participants were included in the sample. The final sample consisted of 20 children (13 female) and 29 adults (24 female). The children ranged in age from 10 to 12 years (mean 11.4, *SD* 0.6); the adults ranged in age from 18 to 32 years (mean 20.4, *SD* 3.3). The children were recruited from a primary school in Melbourne, Victoria. The adults were recruited from the University of Melbourne first year cohort of Psychology students via a Research Experience Program, for which they received course credit. To ensure that the children could understand the task instructions, their estimated full-scale IQ was calculated using the WISC-IV (Wechsler, 2004); 18 children completed the four subtest assessment using Block Design, Similarities, Digit Span and Coding, whilst two children completed the two subtest assessment using Block Design and Vocabulary. The children’s estimated full scale IQs were calculated using Sattler’s method (Sattler and Dumont, 2004), and all scored above 70 (mean 107, *SD* 9, range 83–119).

The University of Melbourne Human Research Ethics Committee and the Catholic Education Office in the Archdiocese of Melbourne approved the study, in accordance with the 1964 Declaration of Helsinki. Parents and children provided written informed consent prior to each child’s participation in the study. Adult participants provided written informed consent prior to the study.

#### Experimental Task

The modified version of the spatial and temporal orienting task (Coull and Nobre, 1998) used in Study 1 was further modified for Study 2. The space and neutral conditions were the same as per Study 1. In the time condition, the central stimulus was presented in an isochronous sequence (**Figure 1**), indicating that the target was likely to appear soon (500 ms FP) or later (1100 ms FP). For the short FP, the background stimulus display was presented for 100 ms, and then the outline of the central stimulus thickened very slightly for 100 ms. This off/on cycle occurred five times in a row, to indicate that the target was likely to appear soon. For the long FP, the background stimulus display appeared for 100 ms, then a thick outline of the central stimulus appeared for 400 ms. This off/on cycle occurred five times in a row, signaling that the target was going to appear later. In the neutral condition, a slightly thickened outline of the central stimulus appeared once for 100 ms and did not provide any specific information about the likely location or FP of the target. The timing of the FP started with the offset of the cue and, in the case of the time cue, with the last stimulus of the isochronous sequence.

Within a trial, participants were initially exposed to the background stimulus display for 600, 700, 800, 900, or 1000 ms inter-trial interval, which was randomized across trials. During the spatial and neutral conditions, the cue was then presented to participants for 100 ms. During the time condition, the cue was presented in an isochronous sequence for 1 s in total for the short FP trials and 2.5 s in total for the long FP trials. For all Cue conditions the background stimulus then remained unchanged for a delay of either 500 or 1100 ms, after which the target appeared in either the left or right peripheral box for 100 ms. Following target presentation in each trial, the background stimulus display was shown for 1500 ms before the next trial commenced.

For the space and time conditions, 32 valid, 8 invalid, and 4 catch trials (44 trials in total) were presented in each of three consecutive blocks (132 trials per condition). For the neutral condition 16 trials were presented in each of three blocks (48 trials altogether). Prior to each block commencing, participants were informed of the nature of the cue in the upcoming block. Each block lasted for between 2 to 3 min and participants were able to take rest breaks in between blocks. The whole task, with breaks, took approximately 25–30 min.

Participants were provided with a training set of 32 valid trials for the space and time conditions, and 16 trials for the neutral condition, prior to the experimental session. This was to ensure they understood the instructions and to learn the association between the isochronous sequence and the short and long FPs in the time condition. The participants were asked to identify the meaning of each of the cues to ensure understanding of the cues. They were reminded to respond to target detection as quickly as possible.

#### Procedure

The children were tested in a quiet setting at their school. The adults were tested in a quiet testing room in the School of Psychological Sciences at the University of Melbourne.

#### Data Analysis

RTs of less than 100 ms (errors of omission, extremely fast RTs) were excluded from analyses. Any RTs to the catch trials were also excluded. For each participant, the mean RT was calculated per trial type and group means (M) and standard deviations (SDs) for each trial type were calculated. The data were normally distributed.

#### Statistics

Statistical analysis was carried out using IBM SPSS software version 23. The validity effect was investigated with a three-way mixed factorial ANOVA with Group (adults, children) as a between-subjects factor, and Cue type (Space, Time) and Validity (valid, invalid) as within-subjects factors. As in Study 1, only the 500 ms FP trials were analysed. Please refer to **Table 2** for the 1100 ms data. The FP and sequential effects were investigated with a three-way mixed factorial ANOVA involving Group (adults, children), FP of the current trial, i.e., FP(*n*) (500, 1100 ms), and FP of the previous trial, i.e., FP(*n* - 1) (500 and, 1100 ms), on the neutral trials only. The alpha level was set at 0.05 and Bonferroni-adjustments were made for pair-wise comparisons.

### Results

#### Spatial and Temporal Validity Effects

**FIGURE 4:**
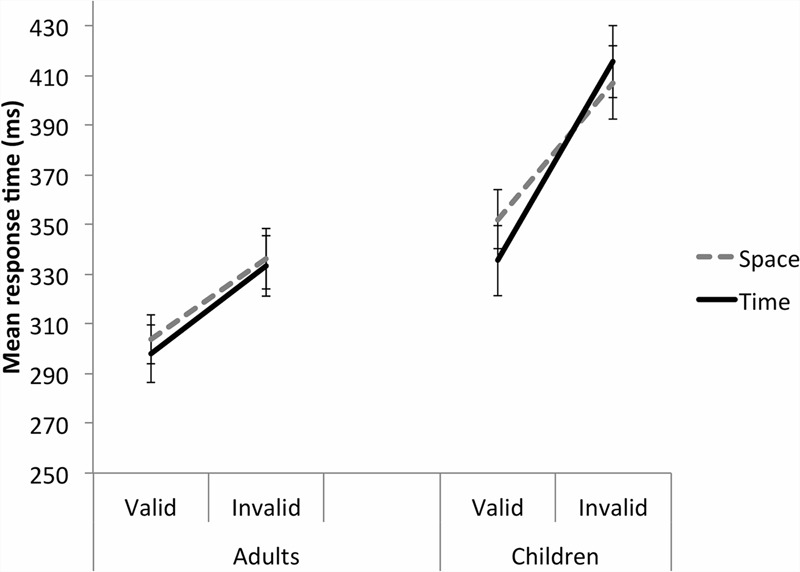
**Study 2 (sequence time cue).** Both the adult and child groups were perturbed by the invalid cues, in both the temporal and spatial dimension, with a greater effect shown by the child group. Error bars reflect standard errors.

Importantly, there were no significant main effects or interactions involving Cue (**Figure 4**; **Table 2**). A Validity main effect, *F*(1,47) = 103.65, *p* < 0.001, $\eta_{p}^{2}$ = 0.688, and a Group main effect, *F*(1,47) = 13.392, *p* = 0.001, $\eta_{p}^{2}$ = 0.222, were further explained by a Validity by Group interaction, *F*(1,47) = 11.341, *p* = 0.002, $\eta_{p}^{2}$ = 0.194. Although both adults and children had significantly slower RTs to targets in the invalid than valid trials, both *p* < 0.001, Cohen’s d adults 0.72, children 0.95, the interaction was most likely driven by the particularly slow responses to invalid trials made by the children, as seen in **Figure 4**.

There were no other significant main or interaction effects.

#### Variable Foreperiod and Sequential Effects

Significant FP(*n*), *F*(1,47) = 23.3, *p* < 0.001, $\eta_{p}^{2}$ = 0.32, and FP(*n* - 1), *F*(1,47) = 32.1, *p* < 0.001, $\eta_{p}^{2}$ = 0.41, main effects were further explained by a significant FP(*n*) by FP(*n* - 1) interaction, *F*(1,47) = 32.17, *p* < 0.001, $\eta_{p}^{2}$ = 0.41 (**Figure 3**; **Table 3**). RTs were significantly slower when the current short FP(*n*) trial was preceded by a long FP(*n* - 1) trial than by a short FP(*n* - 1) trial (*p* < 0.001) – the sequential effect. In contrast, RTs did not vary significantly when a current long FP(*n*) trial was preceded by a long or short FP(*n* - 1) trial, *p* = 0.79 – the asymmetric nature of the sequential effect. When the preceding trial was long, participants responded to the target with significantly faster MRTs at the current trial long FP compared with the short, *p* < 0.001. When the preceding trial was short, there was no significant difference in MRT between the current trial short and long FPs, *p* = 0.884.

There was a significant Group main effect, *F*(1,47) = 6.7, *p* = 0.013, $\eta_{p}^{2}$ = 0.13, with the adults responding significantly more quickly than the children. There were no interactions involving Group.

#### A Direct Comparison of the Validity Effect for the Temporal Cue from Studies 1 and 2

The Validity effect was compared across Studies 1 and 2, to directly test whether the provision of a duration versus a sequential cue was more beneficial in helping children (and adults) to orient attention in time. In terms of age of the two samples, there was no significant main effect of Study, *F*(1,95) = 1.427, *p* = 0.235, $\eta_{p}^{2}$ = 0.015, and no significant interaction between Group (adult, child) and Study (duration, sequence), *F*(1,95) = 0.343, *p* = 0.559, $\eta_{p}^{2}$ = 0.004. By design, there was a significant main effect of Group, *F*(1,95) = 453.080, *p* < 0.001, $\eta_{p}^{2}$ = 0.827.

**FIGURE 5:**
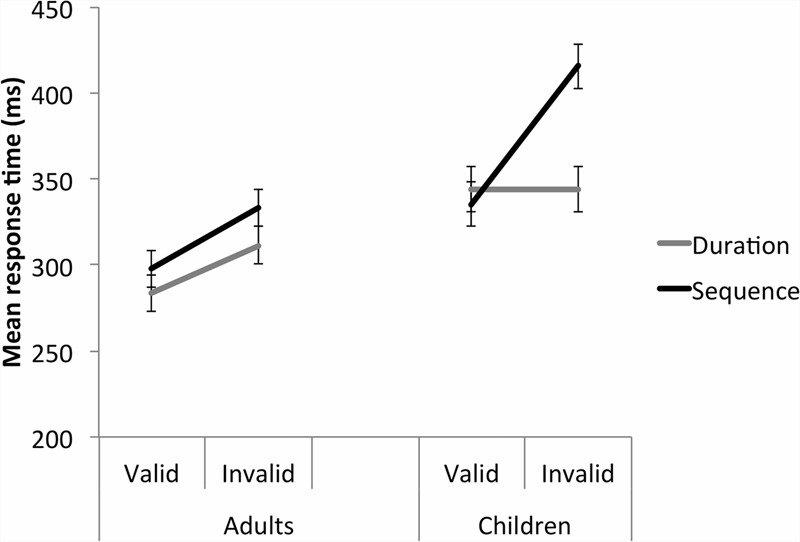
**Studies 1 and 2 – A direct comparison of the time cue trials at 500 ms FP between Studies 1 (duration time cue) and 2 (sequence time cue).** For children, the absence of a response time perturbation by invalid time cues in the Duration study contrasts with a large response time perturbation in the Sequence study. Error bars reflect standard errors. Please note that the time cue data are also represented in **Figures 2** and **4**.

A Group (adult, child) by Study (duration, sequence) by Validity (valid, invalid) three-way repeated measures ANOVA was conducted on the Temporal, 500 ms FP data. Significant Validity, *F*(1,95) = 91.538, *p* < 0.001, $\eta_{p}^{2}$ = 0.491, Study, *F*(1,95) = 4.699, *p* = 0.033, $\eta_{p}^{2}$ = 0.047, and Group, *F*(1,95) = 21.863, *p* < 0.001, $\eta_{p}^{2}$ = 0.187 main effects were further explained by a significant Validity by Study by Group interaction, *F*(1,95) = 23.520, *p* < 0.001, $\eta_{p}^{2}$ = 0.198 (**Figure 5**). This was broken down by Group. For the adults, there was a significant Validity main effect, with significantly faster responses to the valid than invalid trials, *p* < 0.001. There was no Study main effect and no significant Study by Validity interaction. For the children, there was a significant Study by Validity interaction, *F*(1,38) = 35.037, *p* < 0.001, $\eta_{p}^{2}$ = 0.480. For the duration study, there was no significant difference between the valid and invalid temporal trials, *p* = 0.992. For the sequence study, children were significantly faster to respond to the valid compared with invalid trials, *p* < 0.001. For valid trials, there was no significant difference in MRT between the duration and sequence studies, *p* = 0.680. For the invalid trials, in contrast, children in the sequence study were significantly slower to respond to the target than in the duration study, *p* = 0.002.

### Discussion

Supporting our hypothesis, children were able to use the isochronous, visual sequence to orient their attention in time – both children and adults showed the validity effect for spatial and temporal orienting, and indeed the children showed a significanty larger temporal validity effect than the adults. There were no significant effects of Cue, suggesting that the isochronous sequence was as useful as the arrow cue in guiding the orienting of attention in time versus space, both for children and adults. Both children and adults showed the variable FP and asymmetric sequential effects, again supporting previous research. A follow-up analysis of the temporal cue trials only, directly comparing the validity effect of the two studies, confirmed the findings that the provision of the sequential cue aided children (and adults) to orient their attention in time, whereas the duration cue did not offer the same support for the children.

Isochronous sequences have attention-capturing properties (Jones et al., 1982; Breska and Deouell, 2014), orienting attention exogenously to moments in time that are in phase with the entraining rhythm. Notably, however, the rhythm of our sequence did not directly match the timing of the upcoming FP, suggesting that it did not merely rhythmically entrain participants’ attention. The participants had to extract temporal information from the sequence and then extrapolate this information to the forthcoming FP, which was longer (500 ms) than the individual components (100 ms) of the sequence. It is possible, however, that the individual components of the isochronous sequences were combined by participants to produce an interval that was a harmonic of the upcoming FP. For instance, the five 100 ms cues may have been added to the five 100 ms intervals, summing to 1,000 ms, which is double the 500 ms FP. This harmonic may feasibly have helped entrain attention to the upcoming FP. Further research is required to probe this question.

These data replicate previous findings in adults that fast and slow rhythms generate temporal expectancies that a target will appear after a short versus long delay, respectively, e.g., (Barnes and Jones, 2000; Rohenkohl et al., 2011; Sanabria et al., 2011; Breska and Deouell, 2014; Morillon et al., 2014). In this study, we have extended these findings to children aged around 11.

## General Discussion

To select and process important information in the environment requires a flexible cognitive system that can orient attention in both time and space. The exogenous characteristics of the time cue were emphasized firstly by using a duration-based cue, and secondly by using a sequential isochronous cue. The duration cue of Study 1 did not help children to predict target onset in order to speed responding. In contrast, the sequence cue of Study 2 did help children to orient their attention in time and they were strongly perturbed by the invalid time cues. A direct comparison of RTs in Studies 1 and 2 confirmed that sequential, rather than single, presentation of a duration cue was significantly more beneficial for performance. We found that children were able to use the physical temporal properties of sequential presentation to help estimate when the target would appear in the near future. Exaggerating the exogenous nature of the temporal cue therefore helped children orient their attention in time. To our knowledge, this is the first study showing that children can make use of isochronous rhythms to predict the time of target onset in order to speed responses to that target.

Although many prior studies have shown that children (Drake et al., 2000), and even infants (Haith et al., 1988; Colombo and Richman, 2002; Philips-Silver and Trainor, 2005; Werner et al., 2009; Winkler et al., 2009; Brandon and Saffran, 2011), *process* the temporal information inherent in isochronous rhythms, we demonstrate that children can then *use* this temporal information to guide and optimize their responses to temporally predictable events. In adults, the temporal predictability of isochronous or rhythmic sequences orient attention to moments in time that are in phase with the entraining rhythm, thereby optimizing processing of stimuli appearing at those precise moments (Doherty et al., 2005; Martin et al., 2005; Correa and Nobre, 2008; Ellis and Jones, 2010; Rohenkohl et al., 2011; Sanabria et al., 2011; de la Rosa et al., 2012; Miller et al., 2013; Cutanda et al., 2015). In our study, however, the interval before target appearance (500 ms) was not identical to that used in the isochronous sequence (100 ms). Participants had to use a *relatively* short (or long) cue to predict target onset after the relatively shorter (or longer) FP. Sanabria et al. (2011, Experiment 3) have already shown that adult participants can extrapolate the temporal information provided by rhythmic sequences to non-matching FPs (Sanabria et al., 2011). We now confirm this result in children. We suggest that our results do not simply reflect the entraining effects of an isochronous sequence, although it is possible that participants combined the five sequential presentations of the 100 ms stimulus to cue the 500 ms FP. Further research in children, comparing the benefits of isochronous sequences on targets appearing at harmonic versus non-harmonic FPs, will be required to address this possibility.

Many previous studies have shown that multiple, as opposed to single, presentations of stimulus duration sharpen timing in motor and perceptual duration estimation tasks (Schulze, 1989; Drake and Botte, 1993; Merchant et al., 2008; Grondin, 2012). In these studies, the timing benefits of sequential presentation were measured explicitly by the accuracy and variability of duration judgments. We extend these findings in two ways: first by showing that sequential presentation improves timing as measured more implicitly by the speed of RT and, second, by demonstrating this effect in children as well as in adults. Importantly, in these previous studies, timing was improved by sequential presentation whether the test interval was contiguous (in-phase) with the reference rhythm or not (Keele et al., 1989; Ivry and Hazeltine, 1995; Grondin et al., 2001). The temporal benefits of sequential presentation were therefore unlikely to be due simply to entrainment, but rather were interpreted to reflect the construction of a more robust, accurate and less variable temporal template against which the test interval could then be compared. It is therefore possible that in our study, sequential presentation afforded a more temporally robust representation of the short or long cue in memory, which helped children to then orient their attention toward the moment in time at which the (non-contiguous) target was predicted to appear. This point is important because, as opposed to spatial processing, temporal processing depends upon a number of accessory cognitive processes, such as sustained attention and working memory (Michon, 1985; Zakay and Block, 1996; Fortin and Rousseau, 1998). To predict the moment at which the target is expected to appear, the moment of interval onset must be held in working memory and continuously compared to the currently elapsing time until the critical (predicted) time is reached. Children’s timing abilities are known to correlate strongly with mnemonic and attentional capacity (Zélanti and Droit-Volet, 2011, 2012; Droit-Volet, 2013, 2016; Droit-Volet and Zélanti, 2013a,b; Droit-Volet and Coull, 2016) and, as compared to adults, their temporal sensitivity is disproportionally perturbed when their memory of the reference duration is deliberately degraded (Delgado and Droit-Volet, 2007). Therefore, the repeated, sequential presentation of the temporal cue in our study may have provided a robust temporal scaffold to counteract the additional cognitive demands of the temporal task.

In fact, this hypothesis may explain the discrepancy between the results of our own study (Johnson et al., 2015), and that of Mento and Tarantino (Mento and Tarantino, 2015). While we found that children aged 11 could not use an abstract symbolic temporal cue to orient their attention in time (Johnson et al., 2015), Mento and Tarantino (2015) found that children as young as 6 years did benefit from a symbolic temporal cue. One of the main differences between their paradigm and our own was the nature of cue presentation. In the Mento and Tarantino study, the cue remained on the screen until target onset (600 ms short/1400 ms long), whereas in our study it was presented very briefly (100 ms) prior to an empty FP. It may be that the long presentation time of the cue in the Mento and Tarantino (2015) study provided a similarly robust temporal scaffold for interpretation of the cue.

## Conclusion

By combining both exogenous and endogenous stimulus characteristics in the sequential time cue, similar to the combination of exogenous and endogenous features of an arrow space cue (Eimer, 1997; Tipples, 2002; Ristic and Kingstone, 2009; Olk, 2014), we found that children could successfully orient their attention in both time and space. Future research comparing spatial and temporal orienting directly should try to balance the endogenous and/or exogenous characteristics of the temporal cue with that of the spatial cue.

## Author Contributions

Conception and design of the work: KJ and JC. Acquisition, analysis, and interpretation of data: KJ, MB, KP, DD, and JC. Drafting and revision of work: KJ, MB, KP, DD, and JC. Agreement to be accountable for all aspects of the work: KJ, MB, KP, DD, and JC.

## Conflict of Interest Statement

The authors declare that the research was conducted in the absence of any commercial or financial relationships that could be construed as a potential conflict of interest.
